# Mode of Action of Psyllium in Reducing Gas Production from Inulin and its Interaction with Colonic Microbiota: A 24-hour, Randomized, Placebo-Controlled Trial in Healthy Human Volunteers

**DOI:** 10.1016/j.tjnut.2024.12.017

**Published:** 2024-12-26

**Authors:** Alaa T Alhasani, Amisha A Modasia, Mohamed Anodiyil, Maura Corsetti, Abdulsalam I Aliyu, Colin Crooks, Luca Marciani, Joshua Reid, Gleb E Yakubov, Moira Taylor, Amanda Avery, Hannah Harris, Frederick J Warren, Robin C Spiller

**Affiliations:** 1 Nottingham NIHR Biomedical Research Centre and Nottingham Digestive Disease Centre, School of Medicine, University of Nottingham, Nottingham, United Kingdom; 2 Faculty of Health and Rehabilitation Sciences, Princess Nourah Bint Abdul Rahman University, Riyadh, Saudi Arabia; 3 Quadram Institute Bioscience, Norwich Research Park, Norwich, United Kingdom; 4 Food and Biomaterials Laboratory, School of Biosciences, University of Nottingham, Sutton Bonington Campus, Loughborough, United Kingdom; 5 Faculty of Medicine & Health Sciences, University of Nottingham Medical School Queen's Medical Centre, Nottingham, UK

**Keywords:** fermentation, inulin, psyllium, whole gut transit, FODMAPs, microbiota, metagenomics

## Abstract

**Background:**

Recent studies show that the increase in breath hydrogen (BH_2_) and symptoms after ingestion of inulin are reduced by coadministering psyllium (PI).

**Objectives:**

To determine if slowing delivery of inulin to the colon by administering it in divided doses would mimic the effect of PI. Primary endpoint was the BH_2_ area under the curve AUC_0–24 h_. Secondary endpoints included BH_2_ AUC_0–6 h, 6–12 h, and 12–24 h_. Exploratory endpoints included the correlation of BH_2_ AUC_0–24 h_ with dietary fermentable oligo-, di-, monosaccharides, and polyols (FODMAPs) intake and in vitro fermentation results.

**Methods:**

A total of 17 healthy adults were randomly assigned to a single-blind, 3-arm, crossover trial. All consumed 20 g inulin (I) powder dissolved in 500 mL water and mixed with either 20 g maltodextrin (control) or 20 g PI consumed as a single dose or 20 g inulin given in divided doses (DDI), 62.5 mL every 45 min over 6 h. Twenty-four-hour BH_2_, dietary FODMAP intake, stool microbiota, and gas production in vitro were measured. Responders were defined as those whose AUC_0–24 h_ BH_2_ was reduced by PI, whereas nonresponders showed no reduction.

**Results:**

Compared with control, PI did not reduce mean BH_2_ AUC_0–24 h_, whereas DDI increased it, *P <* 0.0002. DDI and PI both significantly reduced BH_2_ AUC_0–6 h_ compared with the control, *P <* 0.0001. However, subsequently, DDI significantly increased BH_2_ from 6 to 12 h (*P <* 0.0001) and overnight (12–24 h) (*P <* 0.0001), whereas PI did so only overnight (*P =* 0.0002). Nonresponders showed greater release of arabinose during in vitro fermentation and higher abundance of 2 species, *Clostridium spp. AM22_11AC* and *Phocaeicola dorei*, which also correlated with BH_2_ production on PI. Dietary FODMAP intake tended to correlate inversely with BH_2_ AUC_0–24 h_ (r = −0.42, *P =* 0.09) and correlated with microbiome community composition.

**Conclusions:**

DDI, like PI, reduces early BH_2_ production. PI acts by delaying transit to the colon but not reducing colonic fermentation over 24 h. Dietary FODMAP intake correlates with BH_2_ response to inulin and the microbiome.

This trial was registered at www.clinicaltrials.gov as NCT05619341.

## Introduction

Fermentable oligo-, di-, monosaccharides, and polyols (FODMAPs) are short-chain carbohydrates that are neither digested nor absorbed in the intestine but fermented by colonic microbiota to produce gases and short-chain fatty acids (SCFAs) [1]. Although SCFAs benefit the human gut, the production of gases has been implicated in the pathophysiology of abdominal symptoms, such as bloating and diarrhea in patients with irritable bowel syndrome (IBS) [2,3]. A low-FODMAP diet has been demonstrated to be effective in treating abdominal symptoms in patients with IBS [1]; however, not all patients respond to a low-FODMAP diet [4].

Two leading hypotheses have been proposed to explain underlying mechanisms. One suggests that the composition of the microbiota determines fermentation products. Several studies have identified microbial signatures of responsiveness [5,6]. The most recent study suggests that those who had a pathogenic microbiota, with significant enrichment of genes involved in lactose metabolism, fructose metabolism, trehalose metabolism, and the biosynthesis of 2 SCFAs (butyrate and propionate), responded better to the low-FODMAP diet [7]. Whether these are associated with gas production is possible but needs further study, with the question being raised of whether stool microbiota is representative enough to predict the response.

An alternative hypothesis is based on MRI studies that have demonstrated that low-molecular-weight FODMAPs (that is, lactose and fructose) increase small bowel water content, whereas higher molecular weight ones, like inulin, have their main effect in the colon [8]. Both types of FODMAPs are fermented anaerobically to produce gases (carbon dioxide, hydrogen, hydrogen sulfide, and methane) [9]. The luminal distension is caused by increased small bowel water and/or bowel gas content, which correlates with the symptoms of gas/flatulence, bloating, pain/discomfort, and diarrhea [10,11]. This hypothesis received further support from the study by Gunn et al. [12], who showed that coadministration of inulin with psyllium (PI) fiber reduces gas production in IBS.

PI is a mucilage derived from the husk of *Plantago ovata* seeds. It forms a highly viscoelastic (“sticky”) gel [13,14] that slows the absorption of both nutrients [15] and water from both the small bowel and the colon [16]. Despite increasing small bowel water, it does not accelerate orocecal transit [17] nor overall gut transit [16] and has been demonstrated to be more effective than control in improving symptoms of patients with IBS [18].

The aim of this mechanistic study was to test the hypothesis that PI’s inhibition of colonic gas production is due to its high viscosity in the small bowel, slowing the delivery of inulin to the colon. We mimicked this without using PI by administering the inulin in divided doses over 6 h. Our aim was to confirm the noninferiority of divided dosage delivery of inulin and PI in achieving a reduction in breath hydrogen (BH_2_) production over 24 h as compared with a control. As our previous studies had shown, the BH_2_ curve had not shown a consistent fall by 6 h [12]; the study period was extended to 24 h, considerably greater than the maximum duration used previously by others (that is, 10 h) [[19], [20], [21]]. To evaluate the possible role of diet and gut transit in mediating the effect of PI and divided dosage inulin, dietary FODMAPs intake and whole gut transit time (WGTT) were measured using the FODMAP calculator and blue muffin test [22], respectively. Furthermore, stool samples were collected from the participants to evaluate microbial community composition and in vitro production of microbial metabolites and to correlate in vivo and in vitro fermentation.

## Methods

### Study design

This was a single-center, randomized, single-blinded, 3-arm crossover trial conducted from October 2022 to December 2022 at Nottingham Digestive Disease Centre, Queen’s Medical Centre, Nottingham, United Kingdom. This study compared the effect of 3 drinks on the production of hydrogen and methane as measured by breath test. The 3 drinks contained 20 g of inulin powder dissolved in 500 mL water. This was administered either as a bolus mixed with 20 g PI husk or 20 g maltodextrin powder (control) or in divided doses of inulin (DDI) (2.5 g/62.5 mL) given every 45 min over 6 h. Maltodextrin has a roughly similar appearance to PI and is known to be rapidly hydrolyzed and absorbed. We have shown in previous studies that it produces minimal changes in small bowel and colon volumes [16].

### Ethical approval

Ethical approval for this study was obtained from the Research Ethics Committee at the Faculty of Medicine and Health Sciences, University of Nottingham (FMHS 17-622).

### Study population

Healthy adults aged 18 y or older and free from gastrointestinal complaints were recruited through poster advertisements on campus at the University of Nottingham. Participants agreed to adhere to a specified dietary and lifestyle restriction, follow a low-FODMAP diet, consume provided meals, and refrain from smoking during the breath test period (see Supplemental information for full details).

### Randomization and blinding

Eligible participants were randomly assigned to their allocated interventions using the http://www.randomization.com platform and were scheduled for a series of 3 study visits, each separated by a washout period of ≥1 wk. Participants could not be blinded to the type of intervention due to the obvious differences between the provided test drinks. To mitigate the risk of bias and ensure single blinding, a team member not involved in breath sample collection, recording, or analysis was responsible for the preparation of the test drinks. Blinding was only broken once data analysis was completed and calculated.

### Study protocol

Subjects were screened for eligibility; demographic data and concomitant medication were recorded (Supplemental Figure 1). Allocation to treatment was randomized, participant flow shown in Supplemental Figure 2. At the screening visit, participants ingested 2 muffins colored with blue food dye (see Supplemental Information for details) and subsequently recorded the time for their stool to go blue as a measure of WGTT [22]. For the in vitro fermentation study, participants were given a stool collection kit and guidelines and asked to collect the samples beforehand and bring them on their first study day before any intervention. They were also asked to complete 4 d (2 weekdays and 2 weekend days) of dietary recall in the 1–2 wk before the first study day using individual access to an online website, “Intake 24” (https://intake24.co.uk/). The collected dietary data were manually transferred by the clinical dietitian to the Monash FODMAP calculator, an online research tool developed by Monash University (https://www.monashfodmapcalculator.com.au/) from which dietary FODMAP contents have been calculated. The generated FODMAPs report included the consumption of simple carbohydrates in grams, including glucose and fructose, and FODMAPs such as excess fructose (free fructose minus free glucose) [23], lactose, polyols (sorbitol and mannitol), fructans, and galacto-oligosaccharides (GOS). Participants also recorded their stool form using the Bristol Stool Form Score (BSFS) of their bowel movements following the study day (see Supplemental information for details).

### Study day

**FIGURE 1:**
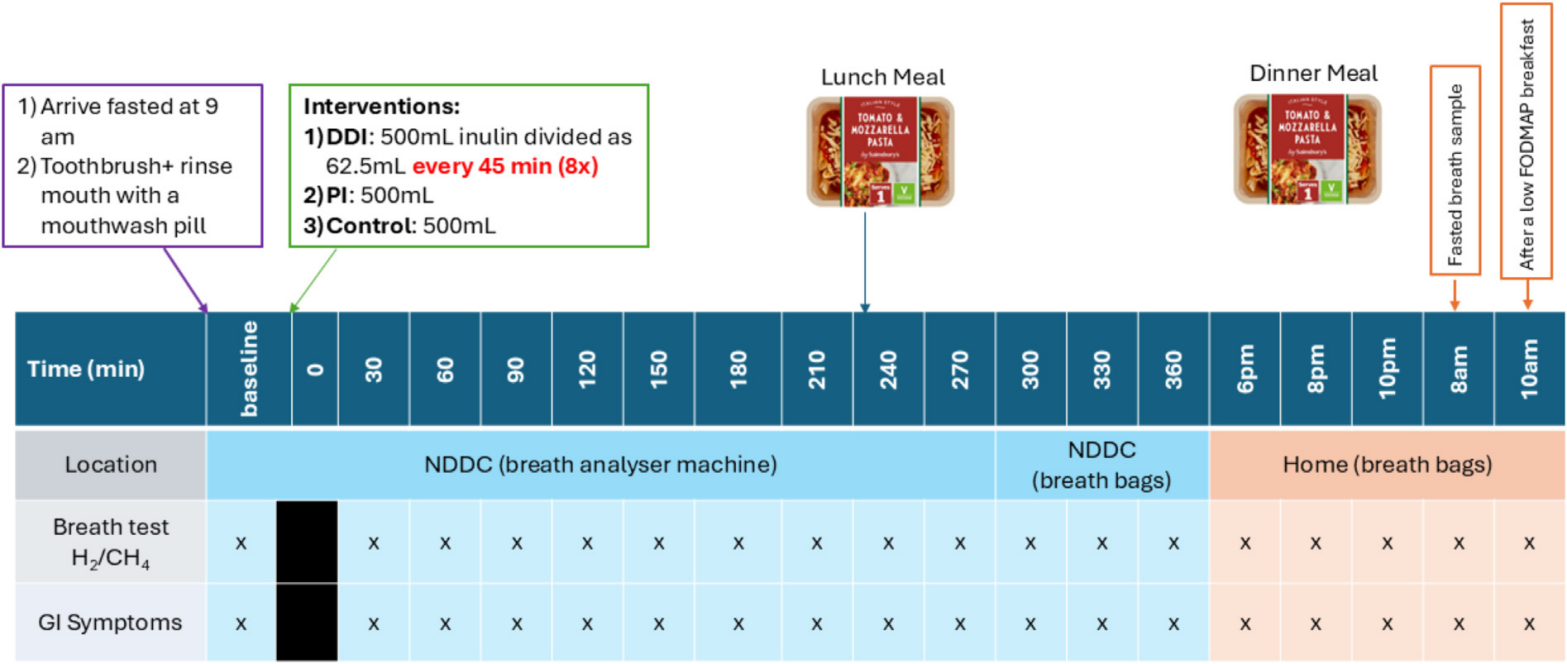
Study day design. Participants arrived fasted and were allocated to one of 3 interventions with regular breath sampling over the 24-h study day. 0–6 h were spent in the study site and the remaining time at the participants’ homes.

Participants came to the study site, fasted, and provided a baseline breath sample after appropriate oral hygiene on site. BH_2_ and breath methane (BCH_4_) were measured using a Gastrogenius-LABORIE breath analyzer machine during the 6 h when the participants were on site and subsequent samples were collected in breath bags (Gastrogenius, Laborie) when participants were at home (Figure 1). Breath collection bags were used to collect the last 3 samples in the study site (at 300 min, 330 min, and 360 min post-treatment intakes) to confirm that they gave the same reading as direct breath test. Subjects were considered methane producers if methane production was ≥5 parts per million (ppm.min.) [24]. After ingesting the test drink (Time 0), breath tests were taken every 30 min for 6 h. A 491-kcal lunch meal of tomato and mozzarella Pasta along with 200 mL water was provided 3.5 h after consuming the test drink. Subjects completed a modified Gastrointestinal Symptoms Rating Scale (GSRS) for gas/flatulence, bloating, abdominal pain, diarrhea, or loose stool using a score from 0 to 3 [25] (see Supplemental information for details).

Responders were defined as participants whose AUC_0–24 h_ BH_2_ was reduced by PI compared with the control. Nonresponders are defined as those whose AUC_0–24 h_ BH_2_ was increased or unchanged by PI compared with the control.

### Interventions

The carbohydrates utilized in this study included PI husk (Supernutrients, Bath, United Kingdom), inulin (Orafti®HP, sourced from Beneo), with a degree of polymerization of ≥23, and maltodextrin (GLUCIDEX® 2, provided by Roquette UK Ltd). Details of preparation are given in Supplemental information and Supplemental Figure 3.

### In vitro fermentation study

To assess the kinetics of fermentation, an in vitro batch fermentation model was set up to explore gas production of inulin fermentation in the presence or absence of PI. Fermentation vessels were seeded with fecal samples from the human study. Gas production from the 2 test fibers (inulin and PI) was measured using the well-established single-stage anaerobic colon models [26] using the ANKOM RF gas production system (ANKOM Technology). Gas production from the test substrates was calculated using previous methods [27]. The data are reported as the cumulative gas volume (mL) produced during fermentation from 0–24 h. ^1^H-NMR was used to assess the concentrations (μM) of end products of microbial fermentation in vitro. Supplemental information provides further technical details.

### Microbiota genomics and metabolomics

Microbial profiling of the fecal samples was performed using shotgun metagenomic sequencing (Illumina NextSeq500) (see the Supplemental information and Supplemental Table 1 for full details).

### Endpoints

The primary endpoint was the AUC of the total BH_2_ over 24 h (BH_2_ AUC_0–24 h_).

The secondary endpoints were: *1*) AUC of the BH_2_ over the first 6, 6–12, and 12–24 h (BH_2_ AUC_0–6 h, 6–12 h, and 12–24 h)_ ; *2*) AUC of the BCH_4_ over 24 h (BCH_4_ AUC_0–24 h_); and *3*) dietary FODMAPs intake.

The exploratory endpoints were: *1*) WGTT; *2*) scores of the symptoms of gas/flatulence, bloating, abdominal pain, and diarrhea or loose stool; *3*) BSFS of bowel movements after intervention intake; *4*) differences in characteristics between “responders” and “nonresponders;” *5*) taxonomic profiling of gut microbiome differences between responders and nonresponders; *6*) effect of habitual dietary FODMAPs intake on BH_2_ response to inulin; and *7*) redundancy analysis (RDA) of the impact of FODMAPs intake on the gut microbiome.

### Statistical analysis of in vivo data

The study aimed to test whether the reductions in BH_2_ AUC resulting from administering inulin in divided doses are noninferior to that seen with coadministering PI, both being compared with maltodextrin control. From previous studies, PI reduces BH_2_ compared with maltodextrin between 290 and 360 min by 1193 ppm.min with a standard deviation of 896 ppm.min. The divided dose regimen was judged to be noninferior to the PI if it reduced BH_2_ AUC _290–360 min_ by ≥350 ppm.min compared with control (that is, a delta of 840 ppm.min. compared with the PI).

To detect this with 80% power and alpha 0.05, 15 subjects were required to be randomly assigned to 1 of 6 different sequences of the treatments (balanced for sequence and period using a Latin square). Although the study design was not identical owing to the different durations (6 compared with 24 h), the previous study data provided the best estimate of the numbers needed. To allow for dropouts and technical failures, we aimed to recruit an additional 3 people to give an overall total of 18 people.

All statistical analyses were conducted using GraphPad Prism V.9. All data tested for normality, and the normally distributed data were expressed as mean ± SD and as the median ± IQR for non-normally distributed data. An additional analysis of the difference in BH_2_ AUC between the treatment groups with an interaction with the time intervals of 0–6, 6–12, and 12–24 h was analyzed with a mixed effects model, including a random intercept for participants (using the lme4 function in R version 4.4.0). Although the distribution of breath hydrogen was skewed, the within patient differences being tested between the treatment groups were normally distributed and met the model assumptions.

### Bioinformatic analysis

Linear modeling was applied to identify taxa which were associated with WGTT and which were differentially abundant between responders and nonresponders to PI treatment using the MaAslin2 package [28], by fitting the following expressions; expr ∼ WGTT for modeling WGTT and expr ∼ responder for modeling responder status. After prevalence filtering, a total of 194 taxa were included in the model. Each taxon was independently modeled. The data were normalized (total sum scaling normalization), and log transformed before analysis. The LM linear modeling method was applied with Benjamini–Hochberg correction for multiple comparisons. A false discovery rate-corrected *P* value of 0.05 was considered statistically significant. Redundancy analysis was carried out using the MicroViz 0.12.1 package [29]. The data were center log ratio normalized and analyzed by Principal Component Analysis using FODMAPs intakes as constraining variables.

## Results

A total of 17 subjects completed all study arms and were included in data analysis (See Supplemental Figure 2). Most participants were female, and the median age of included subjects was 23 y. All included participants were healthy and nonsmokers, except one (Table 1).

**TABLE 1:** Demographic characteristics and dietary intake data of included participants.

	Participants (*n =* 17)
Demographic characteristics	
**Age (y)**	23 (19–37)
**Gender: male/female**	5 (29%)/12 (71%)
**Weight (kg)**	65.4 ± 12.8
**Height (m)**	1.6 ± 0.11
**BMI (kg/m**^**2**^**)**	24.35 ± 2.95
**Smoking status**	No = 16 (94%); Yes = 1 (6%)
Dietary data	
**Energy intake (kcal)**	1676.6 ± 786.3
**Englyst fiber (g)**	11.1 ± 7.3
**AOAC fiber (g)**	15.8 ± 12.6
**Total FODMAP (g)**	26.2 ± 11.7
**Lactose (g)**	16.2 ± 9.2
**Excess fructose (g)**	2.4 ± 2.6
**Sorbitol (g)**	0.5 ± 0.5
**Mannitol (g)**	0.11 ± 0.02
**Fructans (g)**	6 ± 2.95
**GOS (g)**	1.1 ± 0.6

Data presented as mean ± SD except for age, which is the median (range) and gender (%).

Abbreviations: AOAC, Association of Analytical Chemists; FODMAP, fermentable oligo-, di-, monosaccharide, and polyol; GOS, galacto-oligosaccharides.

### Primary endpoint

#### AUC of the breath hydrogen over 24 h

**FIGURE 2:**
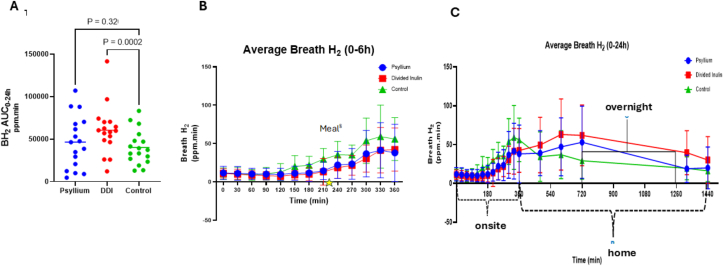
Breath hydrogen time profile. (A) A total AUC_0–24 h_ breath hydrogen for 3 interventions in ppm.min. Divided dose inulin gave significantly higher values compared with control, *P <* 0.0002. Horizontal lines indicate the median. (B) and (C) Mean ± SD of breath hydrogen (ppm) over time for the 3 different drinks over 24 h. The expanded chart shows the first 6 h (B). In (C), (onsite) indicates breath samples collected onsite (0–6 h), (home) indicates breath samples collected at home using breath bags (7–24 h), and (overnight) indicates sleeping time. In (B), ^§^Tomato and Mozzarella pasta meal was provided at 3 h 35 min after the intervention.

The total BH_2_ AUC over 24 h (ppm.min.) was greater after DDI (60,535 ± 28,951) compared with both PI (46,511 ± 30,889) and control (40,306 ± 20,309) (Figure 2). There was an increase of BH2 AUC_0–24 h_ compared with control for both DDI and PI, so it was not appropriate to test our primary endpoint of noninferiority of DDI in lowering BH2 as compared with PI. Post hoc analysis using repeated measures one-way analysis of variance (ANOVA) showed BH_2_ AUC_0–24 h_ after DDI was significantly higher compared with control, *P <* 0.0002, but not to PI, *P =* 0.32.

### Secondary endpoints

#### AUC of the breath hydrogen over the first 6 h

Figure 2 shows the time profile indicating that in the first 6 h, both PI and DDI appear to shift the curve to the right, significantly lowering BH_2_ as compared with control.

The linear mixed effects model with an interaction between time period and treatment, comparing the individual BH_2_ results between the 3 time periods 0–6, 6–12, and 12–24 h, with a random intercept for participants, was used to test the interactions between time and treatment (Table 2). This confirms that both PI and DDI reduce BH_2_ 0–6 h postintervention but that DDI increases BH_2_ both during the evening (6–12 h) and overnight, whereas PI only increases it overnight. If we just compare the AUC _0–6 h_ values, the difference compared with control for DDI was −3771(−5385 to −2157) ppm.min, which, using the a priori threshold for the primary endpoint of a reduction of ≥−350, was noninferior to PI [−3116 (−5585 to −646)].

**TABLE 2:** Time-dependent effects on breath hydrogen production after all interventions.

Time	Average BH_2_ AUC over different time periods ppm.min (mean ± SD)	Liner mixed effects model with interactions between time and treatment, including participants random-effects			
	DDI	PI	Control	DDI compared with control in each time period	PI compared with control in each time period
**0–6 h**	6199 ± 2889	6855 ± 5138	9971 ± 3103	−10.86 (−15.18 to −6.54, *P* < 0.0001)	−8.99 (−13.33 to −4.64, *P* < 0.0001)
**6–12 h**	19736 ± 12564	15784 ± 11849	13669 ± 9361	27.93 (16.2 to 39.66, *P* < 0.0001)	10.25 (−1.64 to 22.14, *P* = 0.0911)
**12–24 h**	42088 ± 21403	29876 ± 20999	20629 ± 12838	35.61 (23.88 to 47.33, *P* < 0.0001)	22.49 (10.59 to 34.38, *P* = 0.0002)

Abbreviations: DDI, divided dose inulin; PI, psyllium + inulin.

#### AUC of the breath methane over 24 h

Only 4 subjects were methane producers, and the BCH_4_ profile over time showed no consistent rise after inulin. The average AUC_0–24 h_ for BCH_4_ was 10722, 5198, and 4088 ppm.min after control, DDI, and PI, respectively, but the numbers were too small for statistical analysis.

### Exploratory endpoints

#### WGTT, GSRS symptoms, and BSFS

The average WGTT [median (IQR)] was 22 h (16.5 – 28.25, *n =* 17). WGTT was found to be significantly correlated with 2 species: *Parabacteroides merdae* and *Blautia wexlerae* (Supplemental Figure 4). Methane producers tended to have a slower WGTT than nonmethane producers, but this was not significant (26.25 h (IQR: 23.5–42.1, *n =* 4) compared with 17 h (IQR, 14–27, *n =* 13); *P =* 0.2).

All tested interventions were well tolerated without any GSRS symptom scores being raised above 1 (mild). The average Bristol score of the first stool passed after PI, DDI, and control did not differ, being 3.7 (range: 2.3–5.5), 3.5 (range: 2.5–5), and 3.4 (range: 1.9–5), respectively. Abnormally hard stool (BSFS = 1 or 2) was reported in 6 subjects after control, 4 after PI, and 4 after DDI, and loose stool (BSFS = 6 or 7) in 1, 1, and 2, respectively.

#### Differences in characteristics of responders and nonresponders to psyllium

Subdividing participants according to their response to PI over 24 h gave 10 responders and 7 nonresponders. Most responders for AUC_0–24 h_ were also responders for AUC_0-6 h_, which correlated strongly with AUC_0–24 h_, r = 0.71, *P =* 0.004. Nonresponders had a significantly higher AUC_0–24 h_ BH_2,_ median (range) of 70,380 (47,610–107,055), whereas responders were lower at 25,050 (4725–67,890), *P =* 0.0007, Mann–Whitney test. There was no significant difference between the groups in terms of age and BMI. Responders tended to have a slower mean WGTT (29.1 (15.8) h) than nonresponders (17.3 (7.1) h), but this was not significant (*P =* 0.073). Total FODMAP intake in responders [median (IQR)] was 29.1 (14.5–41) g, not significantly different from nonresponders intake, which was 23.1 (15.9–33.4) g (*P =* 0.54) (Table 3).

**TABLE 3:** Differences between responders and nonresponders to psyllium effect.

	Age (y)	BMI (kg/m^2^)	WGTT (h)	Psyllium BH_2_ AUC (0–6) (ppm.min)	Psyllium BH_2_ AUC (0–24) (ppm.min)	FODMAP (g)
**Responders to psyllium (*n =* 10)**	21.5 (20–26.8)	24.42 ± 2.1	24 (17–33.5)	3788 (1688–5546)	25050 (4725–67890)	29.1 (14.5–41)
**Nonresponders to psyllium (*n =* 7)**	29 (19–26)	24.14 ± 4.1	17 (12–25)	11280 (6315–15540)	70380 (47610–107055)	23.1 (15.9–33.4)
***P* value for difference**	0.29	0.85	0.073	0.02	0.0007	0.54

Data presented as median (IQR), except for BMI presented as mean ± SD.

#### In vitro fermentation

**FIGURE 3:**
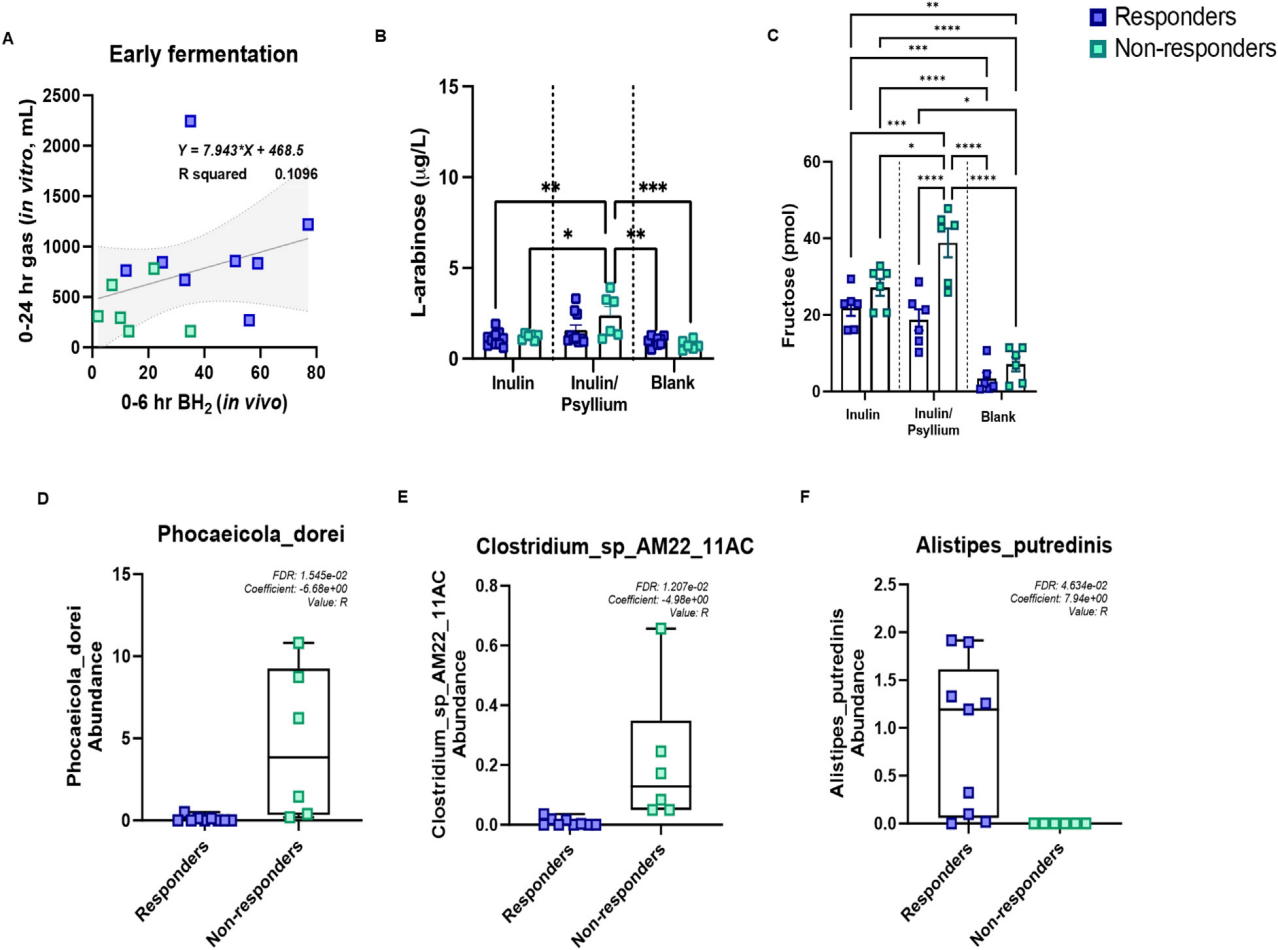
In vitro fermentation. In vitro fermentation revealed differences in fermentation profiles of inulin and psyllium. (A) Correlation between early fermentation using total gas production during 24 h in vitro fermentation and in vivo BH2 production at 6 h. (B) L-arabinose concentrations (μg/L) analyzed in vitro in media after 24 h fermentation of inulin and inulin/psyllium in responders (*n =* 9) and nonresponders (*n =* 6). (C) Fructose concentrations (pmol) analyzed in vitro in media after 24 h fermentation of inulin and inulin/psyllium responders (*n =* 6) and nonresponders (*n =* 6). Statistically significant associations between in vivo BH2 in inulin/psyllium treatment and abundance of *Phocaeicoli dorei* (D), *Clostridium spp. AM22 11AC* (E) and *Alistipes putredinis* (F) between responders (*n =* 9) and nonresponders (*n =* 6). Statistical significance carried out using linear fit model determined using MaAslin2 (*Phocaeicoli dorei*: FDR = 1.545e-02, coefficient: −6.68e + 00; *Clostridium spp. AM22 11AC:* FDR = 1.21e-02, coefficient = 4.98e + 00; *Alistipes putredinis*: FDR = 4.63e-02, coefficient = 7.94e + 00). Statistical significance for (B) and (C) calculated using Mann–Whitney test (GraphPad Prism v10). Values of ∗*P <* 0.05, ∗∗*P <* 0.01, ∗∗∗*P <* 0.001 and were considered statistically significant.

A positive linear correlation was observed between 0 and 24 h gas production of inulin in vitro*,* and between 0 and 6 h BH_2_ production in vivo (R_2_ = 0.11) (Figure 3A). Significantly increased L-arabinose concentrations were detected after 24-h fermentation of PI compared with inulin alone (Figure 3B; 1.214 ± 0.069 compared with 2.376 ± 0.4901 μg/L for inulin and PI, respectively, *P <* 0.05) suggesting that the degradation of PI could occur in vivo. Furthermore, the increased fructose concentrations in nonresponders suggest the enhanced degradation of inulin in the presence of PI.

#### Microbiological differences between responders and nonresponders to psyllium

Degradation products of PI (arabinose) were higher in nonresponders compared with responders, although this did not reach statistical significance (Figure 3B; 2.376 ± 0.4901 compared with 1.586 ± 0.2759 μg/L, respectively). However, the degradation products of inulin (fructose) were significantly higher in nonresponders during combined fermentation of inulin and PI (Figure 3C; 38.81 ± 3.792 compared with 18.71 ± 2.787 pmol in nonresponders and responders, respectively; *P <* 0.0001). Taxonomic profiling using MetaPhlAn4 [30] and MaAsLin2 [28] for species associations revealed 2 species that are more abundant in nonresponders as compared with responders: *P*. *dorei* and *Clostridium* spp. AM22_11AC and one species, which was more abundant in responders, *Alistipes putridinis* (Figure 3D–F). Although associations did not reach statistical significance, *P. dorei* was positively associated with total BH_2_ (over 24 h) after PI treatment [*P* value = 0.0001, q-value (corrected for multiple comparisons) = 0.06, data not shown], whereas *A. putridinis* was negatively associated (*P* value = 0.004, q-value = 0.18). Similarly, *Clostridium* spp. AM22_11AC correlated with BH_2_ (0–6 h) for PI treatment (*P* value = 0.0004, q-value = 0.009), data not shown.

#### Effect of habitual dietary FODMAP intake on breath hydrogen response to inulin

The average FODMAP consumption assessed over 1–2 wk before the first study day was 26.2 g (9.1–46.7). The highest average intake was for lactose (16.2 ± 2.6 g), followed by fructans (6 ± 2.9 g). The lowest intake was for mannitol (0.12 g ± 0.15). The average number of days with valid entry was 3.5 (2–4), and the average number of reported food items was 37.4 (18–62).

**FIGURE 4:**
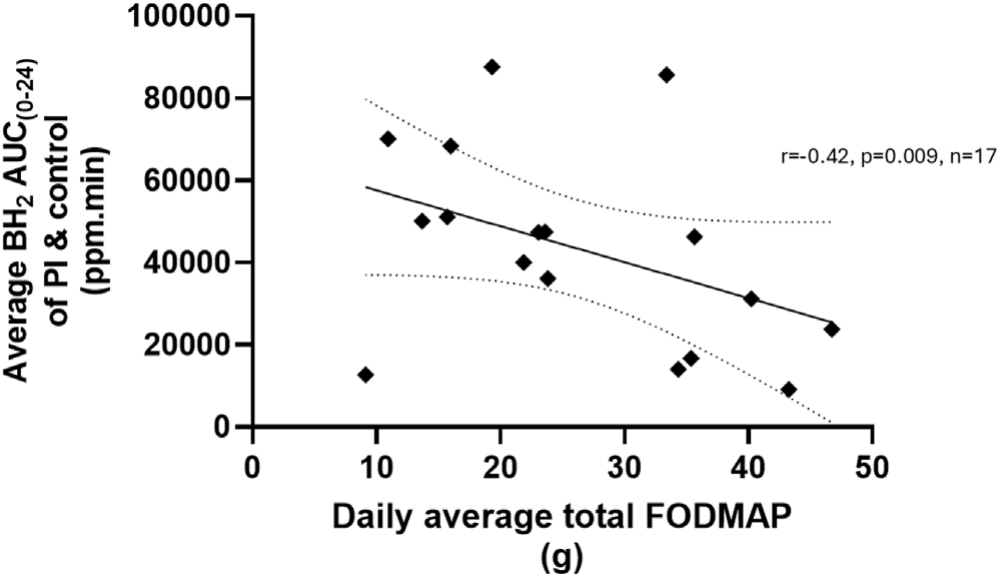
Correlation between 24-h breath hydrogen excretion and daily FODMAP intake. The plot shows the average breath hydrogen excretion over 24 h for PI and control together compared with the daily average total FODMAPs intake. The correlation between breath hydrogen excretion and daily average total FODMAP intake was Pearson *r* = −0.42, *P =* 0.009 (*n =* 17). FODMAP, fermentable oligo-, di-, monosaccharide, and polyol; PI, psyllium.

Since BH_2_ AUC_0–24 h_ after PI and control did not differ, we used the average value in the 2 treatment arms as the best estimate and correlated this with the daily average FODMAP intake (Figure 4), Pearson *r* = −0.42, *P =* 0.009, *n =* 17.

#### Microbiological correlations with dietary FODMAP intake

**FIGURE 5:**
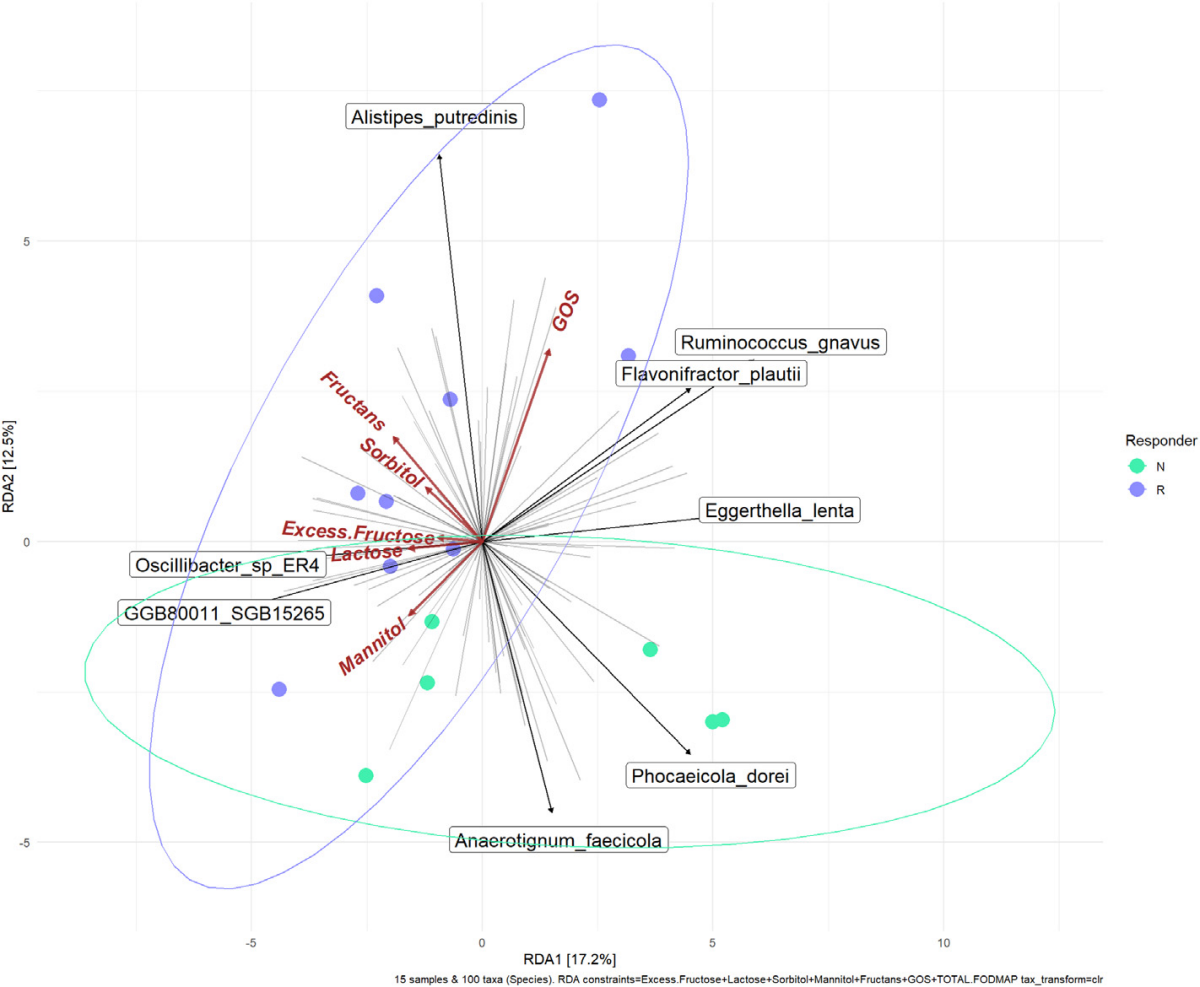
Redundancy analysis (RDA) plot showing the association between dietary intake of FODMAP_S_, gut microbiome composition at baseline and responder status to the psyllium intervention. Responder status is indicated by color, Responder = •, and Nonresponder = • , with the ellipses representing 95% confidence level. Responder status was aligned with FODMAP intake, whereas bacterial species were correlated both positively and negatively with FODMAP intake. FODMAPs, fermentable oligo-, di-, monosaccharides, and polyols.

The RDA shows a clear separation between the responders and nonresponders to the PI treatment (Figure 5), with the responders being associated with a higher intake of FODMAPs. The microbiome associations (Figure 5 and Supplemental Figure 5) observed suggest that microbiome differences associated with FODMAPs intake may lead to differences in response to PI treatment.

#### Effect of psyllium on SCFA production during in vitro fermentation

**FIGURE 6:**
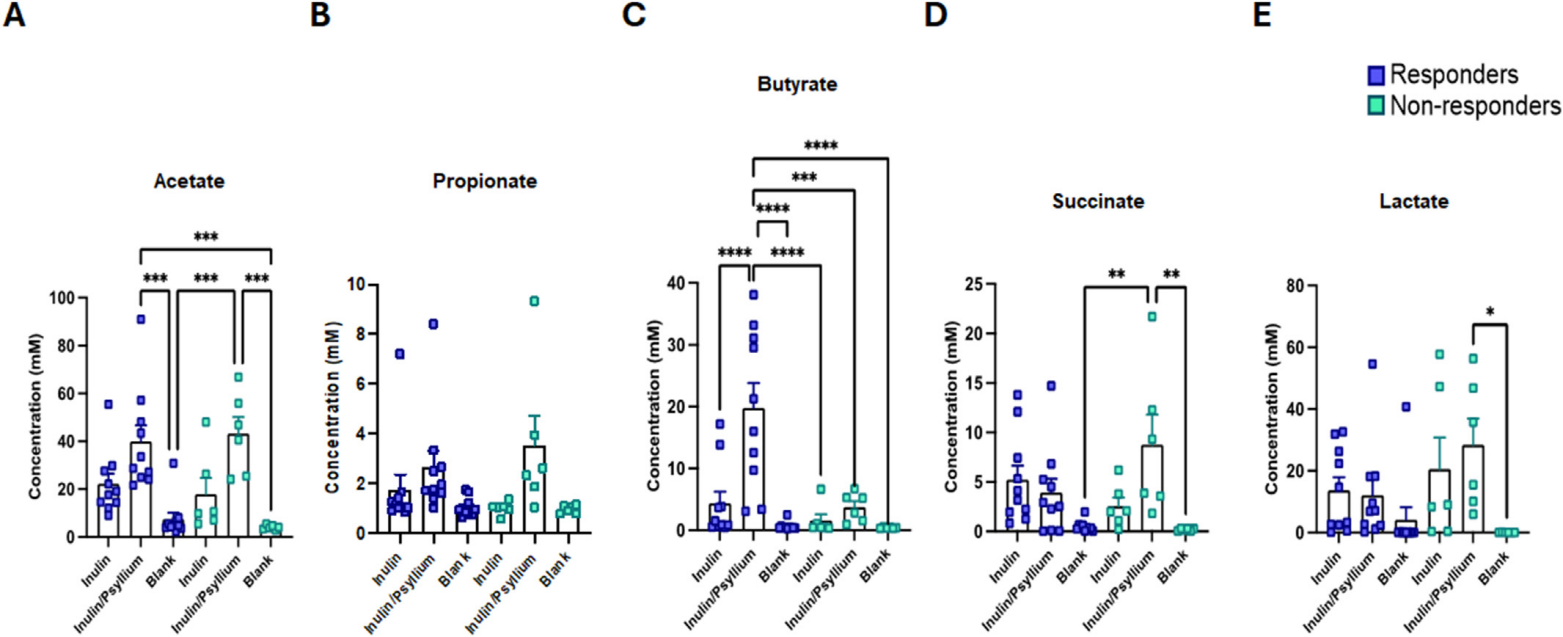
SCFA production during 24 h in vitro fermentation. (A) Acetate, (B) propionate, (C) butyrate, (D) succinate, and (E) lactate concentrations (mM) in media analyzed after 24-h fermentation of inulin and inulin + psyllium using 1H NMR in responders (*n =* 10) and nonresponders (*n* = 6). Analyzing all samples together showed psyllium significantly increased acetate, propionate, and butyrate with no significant change in succinate or lactate. Considering responders and nonresponders separately showed the same effects except for butyrate, which was only increased in responders (*P =* 0.05). Statistical significance calculated using unpaired t-test (GraphPad Prism v10). Values of ∗*P <* 0.05, ∗∗*P <* 0.01, and ∗∗∗*P <* 0.001 were considered statistically significant. SCFA, short-chain fatty acid.

As shown in Figure 6, adding PI increases butyrate production, but this was only significant for responders. Although SCFA production from inulin correlated as expected with gas production, this relationship was not seen when PI was added (Supplemental Figure 6). Furthermore, there was a different SCFA profile with a trend toward, albeit not significant, enhanced succinate production, suggesting alteration of metabolic pathways.

## Discussion

We hypothesized that slowing the delivery of inulin to the ascending colon would alter metabolic activity to produce less hydrogen gas. Prior studies indicated that inulin passes rapidly through the small bowel reaching the ascending colon within 2–3 h, where it rapidly ferments, producing colonic gas, which leads to the increase in BH_2_ [8]. The large dose of inulin is likely to overwhelm the ability of anaerobic organisms to regenerate reduced nicotinamide adenine dinucleotide (NADH), resulting in excess hydrogen production. We reduced inulin delivery to the ascending colon by giving the inulin in divided doses and showed that this resulted in a reduction of BH_2_ from 0 to 6 h that was not inferior to that produced by PI. However, in the ensuing 18 h, PI was associated with lower breath hydrogen compared with the divided dose regime. During this time period, all inulin should have reached the colon, thus suggesting that the effect of PI is more than just slowing delivery. PI could limit the access of microbiota to the inulin trapped in the gel, or it could alter the fermentation pathways to produce less gas [31]. Our earlier MRI studies showed that PI appears to remain as a bolus as it enters the ascending colon with a separation between the PI and other colonic contents, which persists for some hours [32]. Although this appears to delay fermentation, PI did not reduce breath hydrogen excretion over the entire 24-h period, so it seems likely that the gel does ultimately break down, allowing access of the microbiota to the inulin. This fermentation, which occurs in the late evening and overnight, is likely occurring in the transverse and left side of the colon, given that the median total WGTT was 22 h. Shifting fermentation to the left side of the colon is considered desirable [33] since this is where most colon cancers occur. The shift will ensure that the left colon has adequate SCFAs, especially butyrate, which is known to have antineoplastic properties [34].

The exact way PI alters inulin breakdown is unclear. Inulin is a large molecule (molecular weight 3500–10,800 Da depending on the degree of polymerization), which is largely degraded extracellularly to oligofructose by beta-fructosidases, enzymes secreted by *Lactobacilli, Bacteroides*, and *Bifidobacteria* [[35], [36], [37]]. It is known that in *Bifidobacteria*, the smaller molecules are taken intracellularly, where they are metabolized, producing acetic acid and lactic acid via the fructose-6-phosphate shunt. Conversely, *Bacteroides* species produce primarily succinate, which may be converted to propionate [35]. Studies that model the complex communities in the colon using defined species suggest that *P. dorei* is a keystone species in the metabolism of inulin, which is markedly impaired if *P. dorei* is omitted [38]. This enhancement of inulin degradation may explain why *P. dorei*’s presence in our studies is associated with higher total BH_2_. The first steps of inulin fermentation do not produce gas, but subsequent anaerobic degradation of fructose, glucose, and fructans by many other bacteria produces both H_2_ and carbon dioxide. The fact that the breath hydrogen production from 0 to 6 h correlated strongly with total production (0–24 h) supports the idea that slowing initial fermentation could be beneficial in reducing total gas production. In vitro studies show that the carbon source being consumed more slowly results in lower production of lactic acid and an increase in concentration of acetic acid, formic acid, and ethanol [35], although whether this alters net gas production is unclear.

Although the seminal finding that a low-FODMAP diet reduces symptoms of gas and bloating when compared with a high-FODMAP Australian diet [39] has been confirmed through meta-analysis studies [40], recent data indicate that a very restrictive diet could cause significant nutritional deficiencies and negatively impact gut microbiota [41,42].

Our findings suggest that those with lower habitual FODMAPs intake have increased hydrogen production after ingesting inulin associated with a significantly different microbiome. The negative correlation suggests that those with high FODMAP intake have a microbiome that is better adapted to the FODMAP challenge and can direct metabolism in a more energetically efficient route toward SCFA production rather than H_2_ and carbon dioxide. Previous studies have shown that lactulose [43], GOS [44], oligofructose, and inulin [45,46] all increase *Bifidobacteria* and, in the case of GOS [43], reduce the gas production as assessed by BH_2_.

The idea that giving inulin with PI alters the metabolism to reduce gas production without losing the SCFA production is attractive; however, not all subjects respond. We found that “nonresponders” tended to have a faster WGTT, but larger numbers would be needed to confirm that this is not due to chance. Faster transit would favor microbiota capable of rapid saccharolytic metabolism [47], which might favor hydrogen production. We also found greater evidence of PI degradation in the form of greater arabinose released during in vitro fermentation, so “nonresponders” may have a different spectrum of microbiota that can degrade PI more efficiently and faster, thereby limiting its effect. Keystone species that enhance inulin metabolism in consortia include *P*. *dorei* and *Lachnoclostridium clostridioforme* [38]. We found that *P*. *dorei* was more abundant in the stools of nonresponders who produce more gas in vivo after inulin, suggesting that their more active metabolism can overcome the inhibitory effect of PI.

One of the aims of this study was to link in vitro fermentation with in vivo data. We found that the early in vitro fermentation over 0–24 h showed a positive correlation to the early (0–6 h phase) in vivo (Figure 3A), suggesting that this is a better model for predicting in vivo effects than the more usual 0–96-h periods typically used in such studies. We also found that nonresponders had higher numbers of *C. spp. AM22_11AC* and *P. dorei. P. dorei* is well established in the literature as a keystone degrader of insulin and xylans [48]. The lower levels of *P. dorei* associated with high FODMAP intake may contribute to the associated reduction in hydrogen production.

The strengths of this study include the use of a rigorous randomized, placebo-controlled crossover design, well-characterized test materials, and supporting in vitro studies to further define individual fermentation rates, metabolites, and stool microbiota. Weaknesses include the relatively small subject numbers and their heterogeneous response to inulin, which would have required much larger numbers to overcome.

In conclusion, although the early part of the inhibitory effect of PI on inulin fermentation can be mimicked by slowing the arrival of the inulin by giving it in divided doses, there are other effects, as yet unclear, by which PI inhibits gas production compared with divided doses in the later phase of colonic transit (7–24 h postdosing). Not all subjects show the inhibitory effect of PI, which appears to be overcome by adaptive mechanisms within microbiota, including PI degradation. Nonfermentable viscous fibers like methylcellulose may overcome this problem, thus allowing more reliable inhibition of gas production. Furthermore, by improving tolerance, such products could allow increased FODMAP intake with the potential to induce beneficial changes in the microbiome and, in the long term, improve tolerance to inulin.

## Author contributions

The authors’ responsibilities were as follows – ATA, MC, LM, GY, AA, RS: designed research; ATA, AM, MA, AIA: conducted research; ATA, AAM, CC, FW, RS: analyzed data; ATA, AM, MC, FW, RS: wrote the paper; ATA, RS: had primary responsibility for the final content; and all authors: read and approved the final manuscript.

## Data availability

Data described in the manuscript may be made available at a reasonable request. Shotgun metagenomic sequencing data have been deposited in the NCBI SRA and are available under project code PRJNA1109584. For the purposes of peer review, a secure link is available to view the data at the following address: https://dataview.ncbi.nlm.nih.gov/object/PRJNA1109584?reviewer=s5a0f1qjlhbvqimp00qbeu64th.

## Funding

This research was supported by the Medical Research Council Experimental Medicine Project 5954150:I and the Royal Embassy of Saudi Arabia Cultural Bureau, which funded Alaa Alhasani’s PhD. The work of Frederick Warren and Hannah Harris was funded by the Biotechnology and Biological Sciences Research Council (BBSRC); this research was also funded by the BBSRC Institute Strategic Program Food Microbiome and Health BB/X011054/1 and its constituent project BBS/E/QU/230001B.

## Conflict of interest

RS has received research grants from Nestle and Sanofi and is a consultant for EnteroBiotix. MC is consultant for Arena, Biocodex, PROMEDCS, Takeda, Nestle, RB, Mayoly. All other authors report no conflicts of interest.
