# Supplementary material for: Mode of Action of Psyllium in Reducing Gas Production from Inulin and its Interaction with Colonic Microbiota: A 24-hour, Randomized, Placebo-Controlled Trial in Healthy Human Volunteers
Source: J Nutr. 2024 Dec 26;155(3):839–48. doi: 10.1016/j.tjnut.2024.12.017 (PMC11934246; doi:10.1016/j.tjnut.2024.12.017)
Supplement: multimedia component 1 [file mmc1.docx]

# **Supplementary Information**

## **Supplementary Methods**

## **Study protocol**


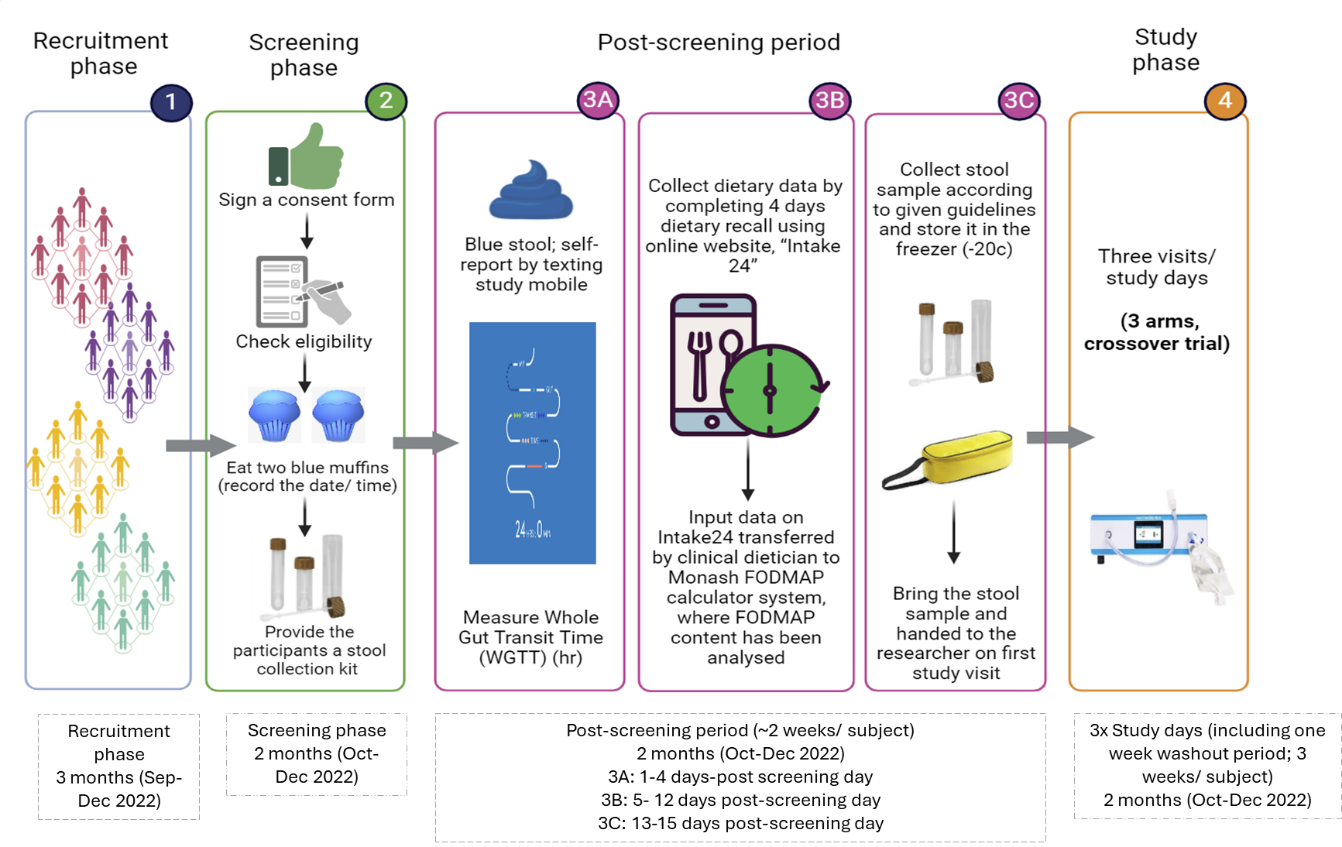
The study included three phases: advertisement and recruitment, a screening visit, a post-screening period, and three study visits (24 hours/visit) (**Supplementary Figure S1**).

**Supplementary Figure S1:** Graphical representation of study phases. The figure was created with Biorender.com

## **Study population**

Exclusion criteria encompassed pregnancy, a history of pre-existing gastrointestinal disorders impacting bowel function (excluding appendectomy), any underlying medical conditions potentially preventing study participation (e.g., diabetes or respiratory diseases), the use of medications known to influence gastrointestinal motility or gut microbiota (e.g., antibiotics, probiotics), night shift work, and individuals assessed by the investigator as unlikely to comply with the study protocol.

## **Dietary and lifestyle restrictions**

Participants were asked to follow a low FODMAP diet for the day before each study day by avoiding foods high in poorly absorbed fermentable carbohydrates (e.g., beans, pulses, and lentils), excessive caffeine-containing drinks (≥3 times), alcohol, probiotics, multivitamins, iron supplements or protein shakes/sports supplements as well as strenuous exercise. Participants were fasted overnight and required to remain nil by mouth on the morning of the study day except to swallow essential medicines with water. The participants received a reminder email before study day to ensure these instructions were followed.

## **Study day**

On the study day, each participant was asked to brush their teeth and attend the study site fasting at 9:00 am. Participants were provided with mouthwash pills to rinse their mouths before taking the baseline breath sample. During the study day, breath hydrogen (BH_2_) and breath methane (BCH_4_) were measured using a Gastrogenius-LABORIE breath analyser machine. Samples were collected in breath bags when participants were at home. If the fasting breath of hydrogen was >30 ppm, participants were asked to repeat mouthwash and the baseline test. If the hydrogen level still exceeded 30 ppm, the participant was given the chance to repeat the test on another day or to withdraw from the study. Following baseline measurement, each participant received an allocated intervention and was asked to consume within 10 minutes. After ingesting the test drink (Time 0), breath tests were taken every 30 minutes for 360 minutes. A 491-kcal lunch meal of Tomato and Mozzarella Pasta along with 200 mL water was provided 215 minutes after consuming the test drink. Breath collection bags were used to collect the last three samples in the study site (at 300 min., 330 min., and 360 min. post-treatment intakes). Each participant was provided with breath bag kits to continue the collection of breath samples at home every 2 hours (at 6:00 p.m., 8:00 p.m., 10:00 p.m., 8:00 a.m. and 10:00 a.m., on the next day) and return these bags to the study site for analysis.

## **Supplementary Results**


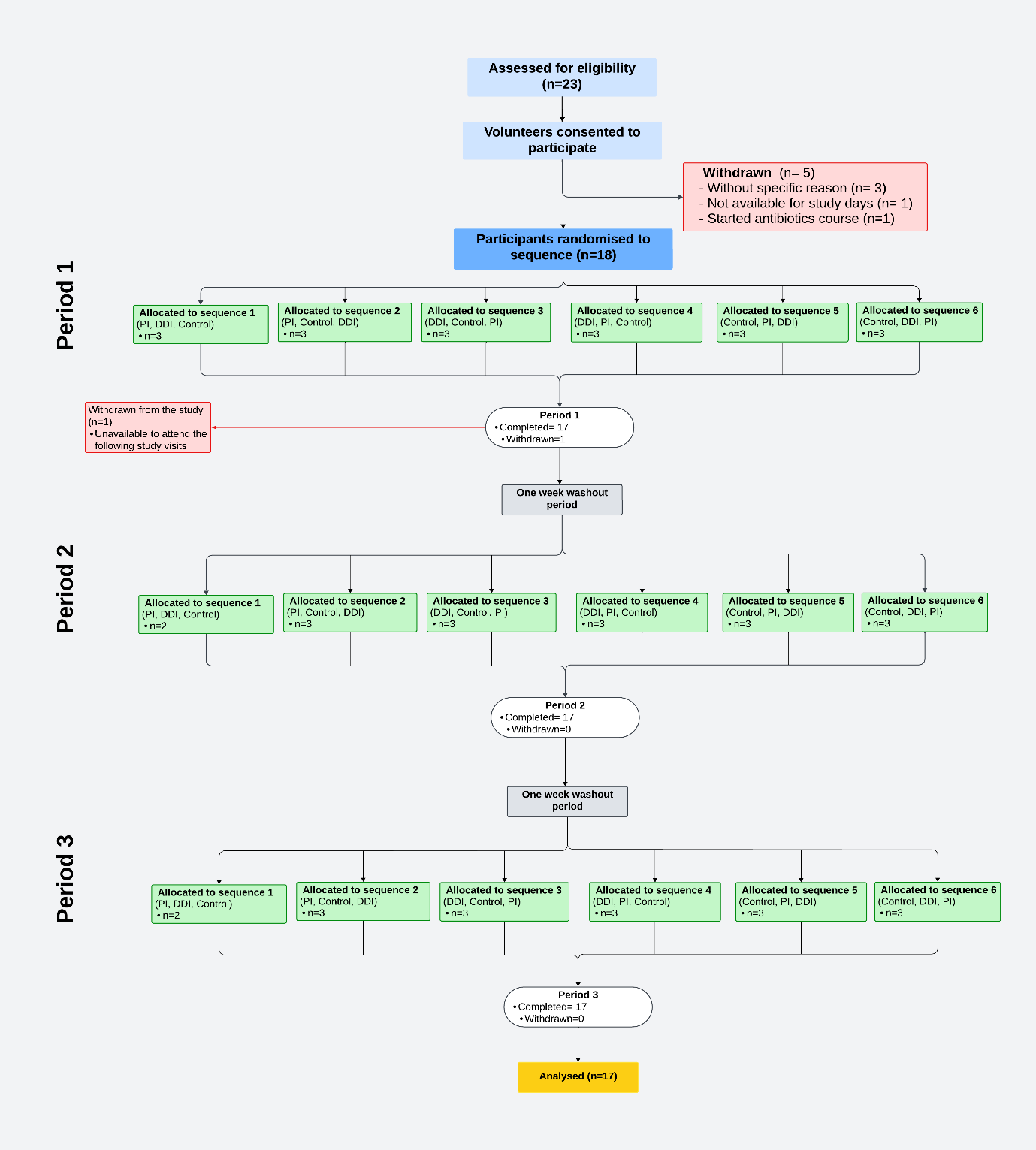


**Supplementary** **Figure S2. Consort figure for recruitment*:*** A total of 23 healthy subjects attended the screening session, and all met the inclusion criteria. After obtaining informed consent,6 subjects withdrew from the study: 4 without giving a reason, one because of tonsillitis, and one dropped out after the first study visit, totalling 17 subjects who completed all study arms.

## **Preparation of test drinks**

The inulin drink was prepared by weighing 20g of inulin powder and dissolving it in 500 mL of boiling still water, which was then cooled in a refrigerator overnight. This solution was used the following morning.

### **Psyllium test drink**

The psyllium (PI) was prepared just before consumption. It involved adding 20g of psyllium husk to 500 mL of a previously prepared inulin solution. The psyllium drink intervention was divided into quarter portions to prevent the drink from gelling in the mouth, which could affect its palatability. Each portion was prepared and consumed within 5 minutes. Each given portion was stirred until well mixed and homogenised before consumption.

### **Control test drink**

The maltodextrin drink was prepared immediately before consumption by adding 20g of maltodextrin to 500 mL of a previously prepared inulin solution. The solution was gently stirred until fully dissolved and used as a control.

### **Divided dose inulin**

The inulin drink prepared as described above and was divided into 8 equal doses of 62.5 mL (2.5 g) given every 45 minutes for 6 hours.

### **Rheology and complex viscosity of psyllium and inulin intervention**

The viscoelastic properties of the psyllium intervention (4 wt% psyllium + 4 wt% inulin in water) was characterised using a MCR 301 rheometer (Anton Paar GmbH, Graz, Austria) equipped with a Peltier temperature control system. Oscillation rheology measurements were performed using a cup (CC27; diameter 28.5 mm, effective depth 40.0 mm) and vane (ST22-4V-40; diameter 22.1 mm, effective depth 40.0 mm) geometry. An amplitude sweep was performed at constant angular frequency (ω = 10 rad s^-1^) and at 37.0°C, with the oscillation strain ranging from 10^-1^ – 10^2^ (%). Measurements were carried out in triplicate, with mean reported values.

We have previously reported on the complex viscosity of this preparation (1); however, we report on a modified preparation of the intervention. For consistency of analysis, the intervention was allowed to equilibrate at ambient temperature for 24 hours to allow for sufficient hydration of the psyllium. The consistency of our previous results with our new data assures that prior preparation of the psyllium intervention provides similar consistencies as if it were prepared immediately before consumption. Further characterisation of the viscoelastic behaviour of the psyllium + inulin intervention are currently ongoing in our lab.


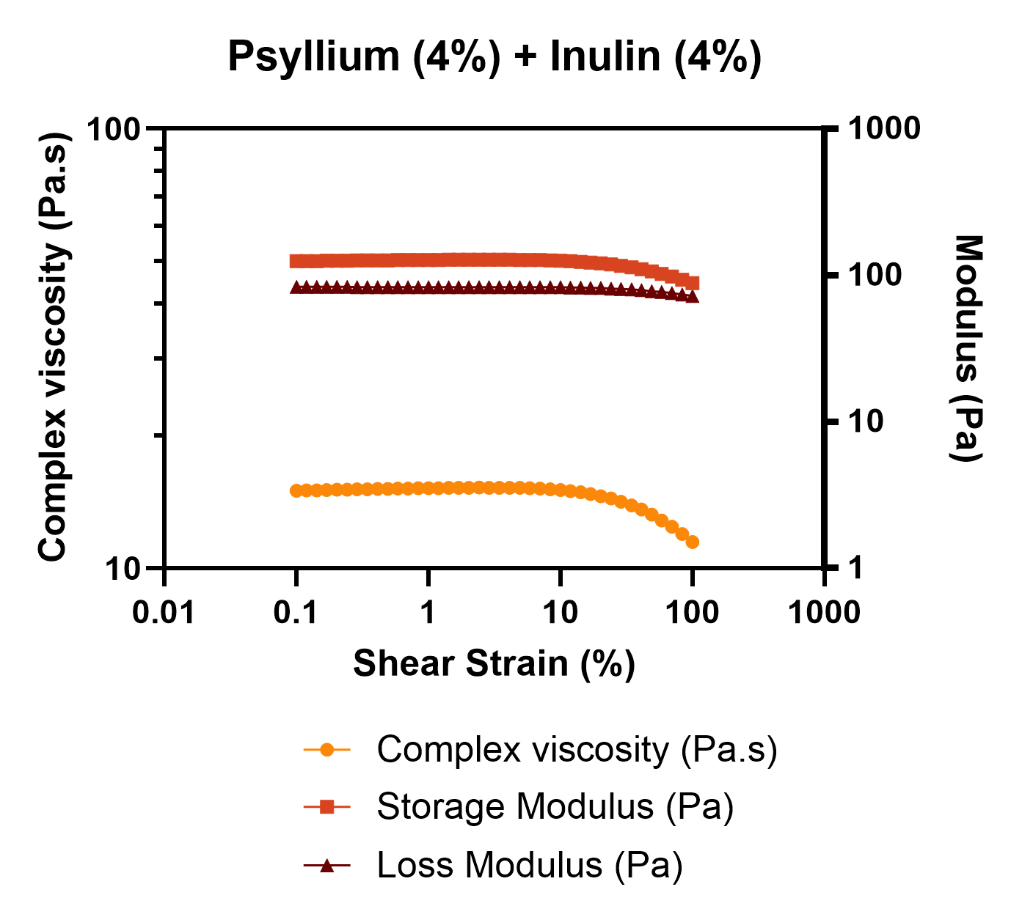


**Supplementary Figure S3**

Complex viscosity (orange circles), storage modulus (red squares) and loss modulus (brown triangles) of the psyllium + inulin intervention as measured using oscillatory rheology (0.1 – 100% oscillation strain). Our values are consistent with our previously reported values for the intervention.^5^

### **Preparation of blue muffins**

Blue-dye muffins were freshly prepared, and the method of preparation, including the ingredients used, as detailed on the ZOE online website (2). The muffin recipe included 245g plain flour, 15g baking powder, 200g granulated sugar, 100g vegetable oil, 230g water, 1 tsp vanilla extract, and 1 tsp concentrated royal blue food dye. To prepare the muffins;

1. Preheat your oven to 170°C (325°F).
2. In a large bowl, mix the dry ingredients (flour, baking powder, and sugar).
3. In another bowl, combine the wet ingredients (oil, water, vanilla extract).
4. Form a well in the centre of the dry mixture and pour in the wet ingredients.
5. Add the blue food dye and whisk until well combined.
6. Divide the batter evenly into 12 muffin cases.
7. Bake for 20-25 minutes or until a toothpick inserted into the centre comes out clean.
8. Let the muffins cool for 20-30 minutes before consuming.

### **Dietary data**

The participants were asked to record their food intake in the 1-2 weeks before the first study using a dietary self-recall online system, “Intake24” (<https://intake24.co.uk/>), for 4 days (2 weekdays and 2 weekend days) dietary recall. Once logged in, the participant is asked to select the meal’s time, type the consumed food and drinks category, and select the dish from various options. After that, using the demonstrative picture, the system asked the participant to determine the meal size and how much was eaten and left over. Finally, the system analysed all dietary input data, converted it into nutrient content values (macro- and micronutrients), and then exported it as an Excel spreadsheet. The FODMAP intake was determined using an online research tool called FODMAP calculator system, developed by Monash University (<https://www.monashfodmapcalculator.com.au/>). The clinical dietician manually transferred the recorded dietary data from Intake24 to the FODMAP calculator system. For single items reported on Intake24, the calculator recorded them as a single item using the same amount (g/mL). For constructed food/recipes, the available recipes on the calculator system were used, and the same amount (g) was used. If the constructed food/recipes were not available on the calculator system, they were created as a new recipe using appropriate guidelines such as McCance & Widdowson's/The Composition of Food (3) and BBC Good food (<https://www.bbcgoodfood.com/recipes>), and then recorded as consumed amount in grams. The completed records were reviewed by the Monash University (FODMAP calculator research group), and then the FODMAP contents of reported food were generated as an Excel sheet. The FODMAP content report included the amount of simple carbohydrates, and FODMAP consumed (in grams) per day, which includes glucose, fructose, free fructose minus free glucose , lactose, sorbitol, mannitol, fructans, galacto-oligosaccharides.

### **Assessment of exploratory endpoints**

Whole Gut Transit Time (WGTT): participants were instructed to consume two blue-dyed muffins within 10 minutes, followed by a cup of water. Subsequently, they were asked to text the study's mobile number or the researcher's email to report the date and time they noticed a blueish discolouration in their stool. The date and time of muffin intake and the observation of blue stool were then used to calculate the WGTT.

Gastrointestinal Symptoms measured by modified Gastrointestinal Symptoms Rating Scale (GSRS) (4) was used to assess the severity of multiple GI symptoms, including gas/ flatulence, bloating, abdominal pain, and diarrhoea or loose stool, using a score from 0 to 3, where 0 means none, 1 = mild, 2 = moderate, and 3 = severe (disabling). These were asked at baseline and after each breath sample (on-site and at home).

Bristol Stool Form Scale (BSFS): each participant was asked to report the consistency (form) of the stool of their first 3-4 bowel movements after intervention intake using a score between 1 and 7; forms 1 and 2 indicated constipation, forms 3, 4, and 5 generally indicated normal stool that easily passes without being too watery, while types 6 and 7 tend toward diarrhoea.

### **Stool sampling**

During the screening session, before any intervention, the participants were provided with a stool collection kit and asked to collect the samples according to the provided guidelines and store them in their home freezer until they brought them to the researcher on their first study visit. These samples were double bagged and frozen at −80°C in a domestic freezer at the Biomedical Research Unit prior to being sent to the Quadram Institute at Norwich Research Park for further analysis of their fermentation potential.

### **In-vitro fermentation study**

Gas production of the two test fibres (inulin and psyllium) was measured using the well-established single-stage anaerobic colon models (5). In brief, per 125 mL vessel, 0.5g inulin or 0.5g inulin and 0.5g psyllium were mixed with 28 mL of media, 3.5 mL of phosphate vitamin and buffer solution, and 0.5 mL of reducing solution as previously described (1, 6) and kept anaerobic under a constant stream of CO_2_. Vessels were sealed and pre-warmed overnight at 37°C prior to inoculation. Faecal samples were thawed in a sealed container in the fridge at 4°C overnight, prior to making a 1:10 dilution in pre-reduced PBS pre-reduced sterile PBS (10 % wt/vol) and homogenised by vortexing with glass beads. For innoculation, 1.5 mL feacal slurry was used, vessels were sealed and incubated at 37°C. Gas production was automatically measured every 5 min using the ANKOM RF system. Gas production from the test substrates was calculated using previous methods (7). The data are reported as the cumulative gas volume (mL) produced during fermentation.

### **Fermentation media and vessel Preparation**

Basal media was prepared as described by (5). Per 125 mL vessel, 38 mL basal solution with 3.5 mL of phosphate vitamin and buffer solution and 0.5 mL of reducing solution were mixed and pH adjusted to 6.8-7. Fermentation bottles contained no substrate (acting as a control to remove any discrepancies in gas produced from sources other than test substrates), inulin (0.5 g) or both inulin and psyllium (0.5 g each). Substrates were mixed and pre-warmed with gentle agitation (~80 rpm) overnight at 37°C.

### **Preparation of faecal sample and inoculation**

Faecal samples were frozen at -20°C prior to use. Faecal samples were thawed in a sealed container in the fridge at 4°C overnight, prior to making a 1:10 dilution in pre-reduced PBS pre-reduced sterile PBS (10 % wt/vol) and homogenised by vortexing with glass beads. The ANKOM system used to measure gas production is a module that screws on top of 100 mL glass vessels we use for fermentation. Once fitted, they provide a sealed anaerobic environment. Once vessels were inoculated, the ANKOM modules were screwed on, vessels were purged with CO2, and then placed in incubator at 37°C. Each substrate was fermented in duplicate per volunteer faecal sample. Bottles were inoculated with 1.5 mL of faecal slurry, sealed to ensure anaerobicity and incubated at 37°C with gentle (~80 rpm) agitation). Gas production was automatically measured every 5 min using the ANKOM RF system. Data are reported as cumulative gas volumes (mL) produced during fermentation from 0-24 hours, averaged from 16 individuals (data from 1 individual was removed due to fault in a vessel) and measured in duplicate per individual/substrate; thus, a total of 32 individual fermentations were performed per substrate.

### **Enzyme assays**

To quantify the extent of inulin and psyllium fermentation, concentrations of fructose and L-arabinose were quantified following 24-hour fermentation in vitro. Samples were collected at 24 hours and centrifuged to remove debris. Samples were stored at -20°C prior to use. Free fructose was measured using a fluorometric assay kit (Abcam, ab 241022) as per manufacturers protocol. Briefly, a 50 µL reaction mix was added to each 96-opaque well plate containing Fructose Standards and samples and incubated at 37°C for 30 min. Fluorescence was measured at Ex/Em 535/587 nm. The background was corrected by subtracting the value of 0 pmol Fructose Standard from all sample readings, and concentrations of fructose in each sample were well calculated from the standard curve. L-arabinose concentrations were quantified using an L-arabinose absorbance assay kit (Megazyme, K-ARGA 04/20) following manufacturers microplate assay procedure. Briefly, a master mix containing buffer and NAD+ was added to each well in 96-well clear flat-bottomed plate. Absorbance (A1) at 340 nm was read after ~3 min, and the reaction started with the addition of 2 µL of β-GalGD/GalM. Absorbance (A2) measured again at the endpoint (~12 min). Absorbance difference (A2-A1) calculated, and concentration of L-arabinose determined using the following calculation: g/L = (ΔA sample/ ΔA standard) x g/L standard x dilution factor.

### **Processing for shotgun metagenomic analysis**

Faecal samples for each individual (n=18) were stored at -20^0^C before use. Samples were thawed at room temperature, and DNA was extracted using a FastDNA™ spin kit for soil (MP Biomedicals). Microbial profiling with the faecal samples from the 18 individuals in the study was performed using shotgun metagenomic sequencing (QIB Illumina NextSeq500).

Briefly, approximately 200 mg of stool was aliquoted, and the remaining procedures were followed according to the recommendations of the manufacturer.

### **Quadram Nextera XT Sequencing Method (NextSeq500)**

Genomic DNA was quantified using the Promega QuantiFluor® dsDNA System (Catalogue No. E2670) and was normalised to 5ng/µl with EB (10mM Tris-HCl). A master mix containing 0.9 µl Tagment DNA Buffer (Illumina Catalogue FC-131-1096), 0.09 µl Tagment DNA Enzyme and 4.01 µl PCR grade water per sample were mixed, and 5 µl aliquoted into each well of a 96 well plate. 2 µl of normalised DNA (10ng total) was pipette mixed with the 5 µl of the tagmentation mix and heated to 55 ⁰C for 10 minutes in a PCR block. A PCR master mix was made up using 10 µl KAPA 2G Fast Hot Start Ready Mix (Merck Catalogue No. KK5601) and 2 µl PCR grade water per sample. 12 µl of this master mix was added to each well to be used in a 96-well plate. 1 µl of 10µM primer mix containing both P7 and P5 Illumina 9bp barcodes (8) (were added to each well. Finally, the 7 µl of Tagmentation mix was added and mixed. The PCR was run at 72⁰C for 3 minutes, 95⁰C for 1 minute, 14 cycles of 95⁰C for 10s, 55⁰C for 20s and 72⁰C for 3 minutes. The libraries were quantified using the Promega QuantiFluor® dsDNA System (Catalogue No. E2670) and run on a GloMax® Discover Microplate Reader. Libraries were pooled following quantification in equal quantities. The final pool was double-SPRI size selected between 0.5 and 0.7X bead volumes using sample purification beads (Illumina® DNA Prep, (M) Tagmentation (96 Samples, IPB), 20060059). The final pool was quantified on a Qubit 3.0 instrument and run on a D5000 ScreenTape (Agilent Catalogue No. 5067-5579) using the Agilent Tapestation 4200 to calculate the final library pool molarity.

The pool was run at a final concentration of 1.5 pM on an Illumina Nextseq500 instrument using a Mid Output Flowcell (NSQ® 500 Mid Output KT v2 (300 cycle) Illumina Catalogue FC-404-2003) for PE150 sequencing following the Illumina recommended denaturation and loading recommendations which included a 1% PhiX spike-in (PhiX Control v3 Illumina Catalogue FC-110-3001). Read depth statistics are show in Supplementary Table 1.

### **Metabolomics**

NMR (Nuclear Magnetic Resonance) buffer was prepared using D_2_O as the solvent and the following concentrations of reagents: 21.7 mM NaH_2_PO_4_, 82.7 mM K_2_HPO_4_, 8.6 mM NaN_3_, 1.0 mM 3-(trimethylsilyl)-propionate-d4 (TMSP). Acetate, propionate, butyrate, succinate, and lactate concentrations were quantified in the contents of *in vitro* bath fermentation vessels following 24-hour fermentation using ^1^H NMR spectroscopy. Briefly, 1 mL of sample from each vessel was collected and centrifuged to remove debris. The supernatant was stored at -20°C prior to use. Samples were thawed and prepared by mixing 400 µL of sample with 200 µL of NMR buffer and added to NMR tubes (Noreu® Standard Series ™, 5 mm). The ^1^H NMR spectrum was acquired for 64 scans, a spectral width of 12,500 Hz and an acquisition time of 2.62 s. Acetate, propionate, butyrate, succinate, and lactate concentrations were quantified using the Chenomx NMR Suite v.

### **Bioinformatics**

The resulting reads were analysed using BioBakery 3 Tools that include Kneaddata for processing and Metagenomic Phylogenetic Analysis (MetaPhlAn) 4 for taxonomic analysis (9). Data visualisation was carried out using the Phyloseq (10) and Phylosmith R package (11). Further microbiome data analysis was conducted using several packages in R, including Microbiome Multivariable Association with Linear Models (MaAslin2) for efficiently determining multivariable associations (12). Redundancy analysis was carried out using the MicroViz R-package (13).

### **Stool Microbiota**

Microbial taxonomic profiles were analysed using MaAsLin2 to determine the multivariable association between WGTT and species abundance. WGTT was found to be significantly correlated with two species, *Parabacteroides merdae* and *Blautia wexlerae* (**Fig. S3A, B**; false discovery rate-adjusted *p*-value; *p*<0.05).


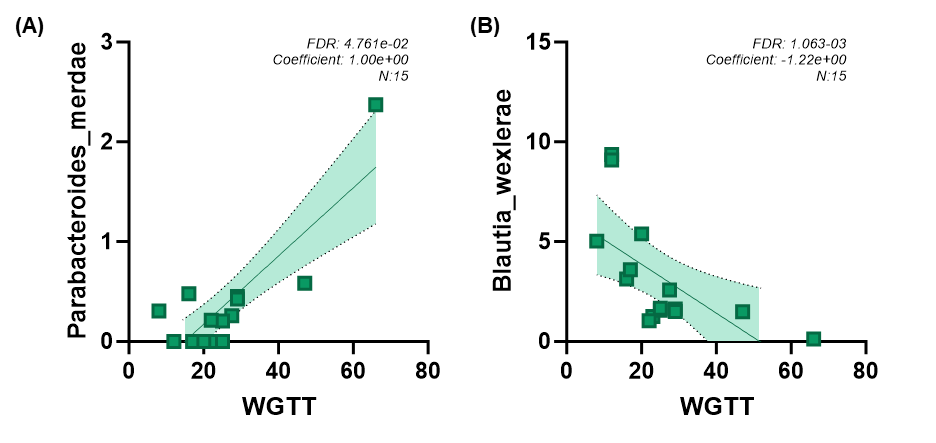
**Supplementary** **Figure S4. Whole gut transit time is correlated to abundance of two species, *Parabacteroides merdae* and *Blautia wexlerae*.** Statistically significant associations between **(A)** *Parabacteroides merdae* (False discovery rate (FDR) = 4.761e-02; n=15) and **(B)** *Blautia_wexlerae* (FDR = 1.063e-03, n=15) were conducted using linear fit modelling determined by MaAslin2.

### **Metagenomic Analysis of Gut Microbiota in Methane Producers**

Only 4 subjects produced methane; however, shotgun metagenomic sequencing analysis conducted on baseline stool samples using MetaphlAn4 confirmed that those participants producing methane had a higher percentage abundance of *Methanobrevibacter smithii*. To further elucidate the contribution of *M. smithii* to the production of CH_4_, differential abundance analysis was conducted based on linear models (Maaslin2; <https://huttenhower.sph.harvard.edu/maaslin/>). This revealed a linear and statistically significant association between abundance of *M. smithii* and amount of CH_4_ produced by participants (data not shown).

**Supplementary Figure S5**. Variable correlation heatmap showing individual associations between gut microbiome composition and dietary intake of FODMAPs. The colours are coded according to Spearman’s correlation coefficient, with asterisks depicting a significant correlation (*p*-value < 0.05).
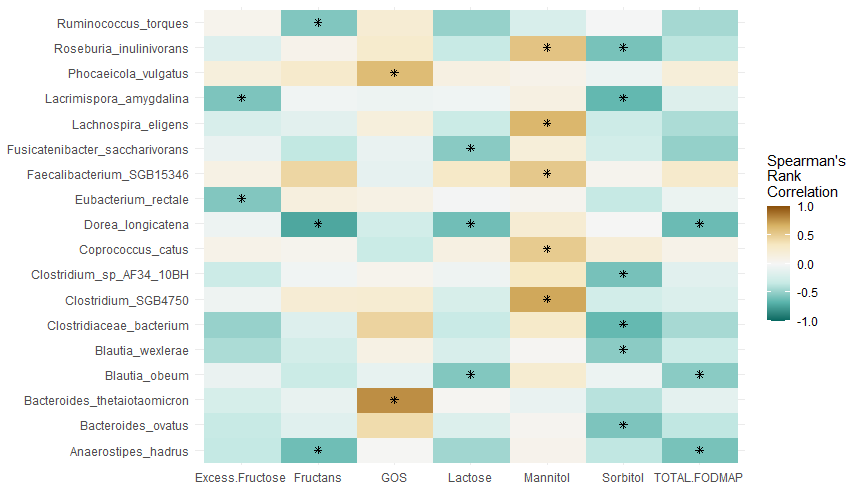


**Supplementary Figure S6**

Correlation between in vitro gas production after 24 hour incubation and SCFA concentrations showing a positive correlation with inulin which is lost when psyllium is added suggesting alteration in metabolic pathways
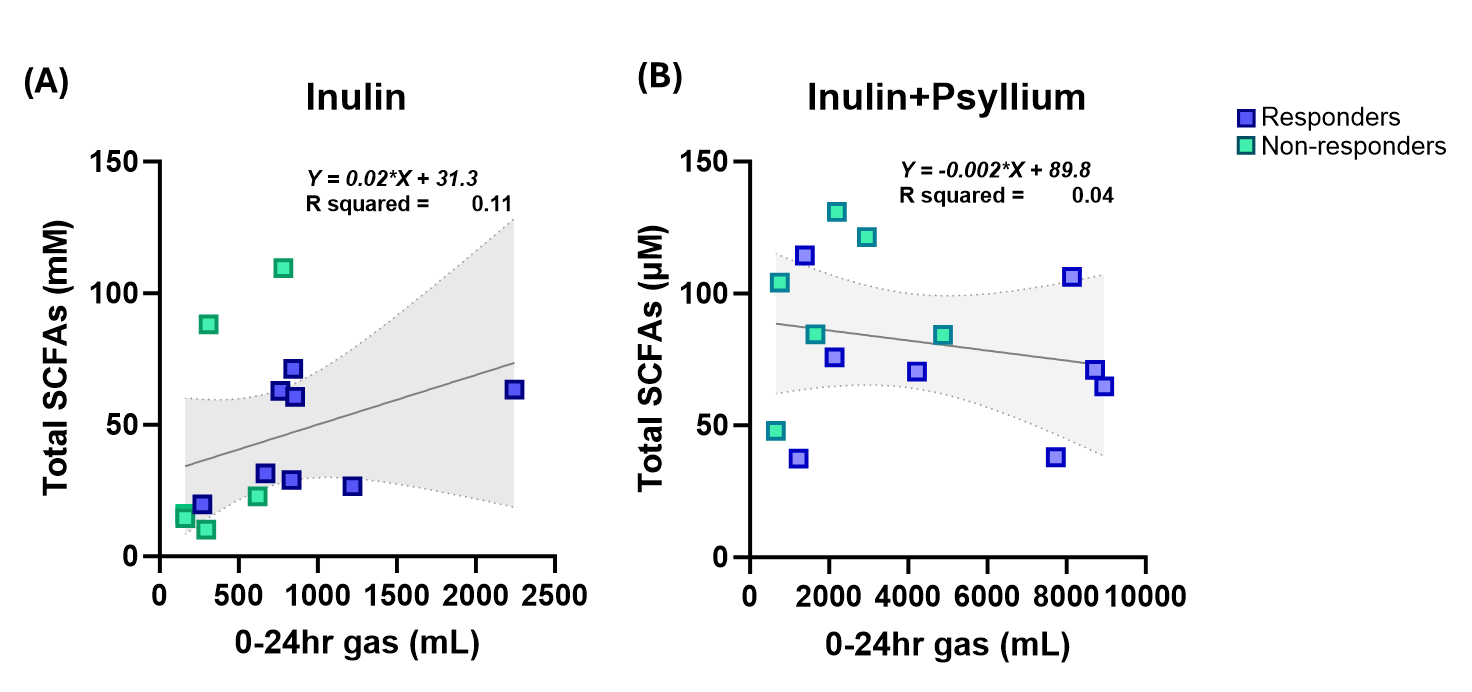


**Supplementary Table S1**

**Table S1 A=raw single – number of raw reads, B=trimmed single – number of trimmed reads, C= decontaminated SILVA_128_LSUParc_SSUParc_ribosomal_RNA single – contaminant reads from bacterial 16S sequences, D= decontaminated hg37dec_v0.1 single – other contaminant reads, E=decontaminated human_hg38_refMrna single – host contaminant reads, F=Final single - final high-quality reads which passed contamination**

| Sample ID | A | B | C | D | E | F |
| --- | --- | --- | --- | --- | --- | --- |
| PID-1421-EON-10_R1_001_kneaddata | 15157255 | 13248591 | 13039140 | 13042218 | 13138666 | 12936695 |
| PID-1421-EON-10_R2_001_kneaddata | 15157255 | 12836702 | 12627365 | 12623431 | 12723671 | 12520766 |
| PID-1421-EON-11_R1_001_kneaddata | 5960416 | 5015347 | 4955473 | 4942239 | 4992955 | 4903247 |
| PID-1421-EON-11_R2_001_kneaddata | 5960416 | 5494143 | 5424405 | 5419741 | 5466761 | 5375853 |
| PID-1421-EON-12_R1_001_kneaddata | 10851866 | 9401415 | 9300317 | 9296772 | 9364408 | 9229379 |
| PID-1421-EON-12_R2_001_kneaddata | 10851866 | 9270179 | 9165819 | 9160668 | 9229303 | 9093874 |
| PID-1421-EON-14_R1_001_kneaddata | 14350452 | 12440010 | 12290749 | 12323054 | 12394118 | 12215556 |
| PID-1421-EON-14_R2_001_kneaddata | 14350452 | 11977251 | 11827789 | 11853896 | 11927064 | 11750478 |
| PID-1421-EON-15_R1_001_kneaddata | 14506153 | 12597547 | 12438433 | 12463227 | 12548284 | 12350333 |
| PID-1421-EON-15_R2_001_kneaddata | 14506153 | 12161578 | 12002110 | 12021108 | 12108178 | 11911980 |
| PID-1421-EON-17_R1_001_kneaddata | 14377655 | 12560141 | 12376300 | 12408555 | 12484898 | 12296777 |
| PID-1421-EON-17_R2_001_kneaddata | 14377655 | 12101465 | 11916908 | 11943602 | 12021709 | 11835669 |
| PID-1421-EON-20_R1_001_kneaddata | 9956335 | 8520979 | 8388282 | 8366398 | 8451365 | 8290023 |
| PID-1421-EON-20_R2_001_kneaddata | 9956335 | 8249256 | 8117544 | 8094698 | 8178748 | 8020605 |
| PID-1421-EON-22_R1_001_kneaddata | 15061893 | 13023394 | 12859965 | 12808822 | 12946259 | 12706777 |
| PID-1421-EON-22_R2_001_kneaddata | 15061893 | 12597932 | 12433139 | 12377350 | 12516171 | 12278852 |
| PID-1421-EON-23_R1_001_kneaddata | 1495419 | 1314323 | 1300241 | 1299608 | 1309000 | 1290525 |
| PID-1421-EON-23_R2_001_kneaddata | 1495419 | 1230135 | 1217057 | 1215946 | 1225254 | 1207411 |
| PID-1421-EON-2_R1_001_kneaddata | 16658039 | 14358821 | 14202709 | 14200214 | 14303771 | 14095606 |
| PID-1421-EON-2_R2_001_kneaddata | 16658039 | 14364826 | 14200524 | 14197118 | 14301959 | 14091860 |
| PID-1421-EON-3_R1_001_kneaddata | 12244567 | 10640891 | 10508769 | 10509380 | 10581862 | 10432876 |
| PID-1421-EON-3_R2_001_kneaddata | 12244567 | 10506527 | 10370007 | 10367687 | 10442233 | 10292003 |
| PID-1421-EON-5_R1_001_kneaddata | 16193567 | 14072447 | 13915138 | 13926232 | 14017449 | 13820497 |
| PID-1421-EON-5_R2_001_kneaddata | 16193567 | 13565952 | 13406303 | 13411766 | 13505221 | 13309238 |
| PID-1421-EON-6_R1_001_kneaddata | 14903872 | 12755889 | 12608045 | 12635207 | 12704140 | 12534781 |
| PID-1421-EON-6_R2_001_kneaddata | 14903872 | 12418052 | 12267334 | 12291758 | 12360755 | 12193887 |
| PID-1421-EON-7_R1_001_kneaddata | 461376 | 405387 | 397113 | 399071 | 401148 | 394945 |
| PID-1421-EON-7_R2_001_kneaddata | 461376 | 394223 | 385960 | 387785 | 389892 | 383752 |
| PID-1421-EON-8_R1_001_kneaddata | 13969788 | 12213571 | 12040500 | 12046031 | 12131553 | 11950073 |
| PID-1421-EON-8_R2_001_kneaddata | 13969788 | 11727417 | 11555808 | 11556725 | 11643223 | 11464311 |
| PID-1421-EON-9_R1_001_kneaddata | 14487073 | 12525806 | 12375718 | 12381057 | 12477163 | 12277191 |
| PID-1421-EON-9_R2_001_kneaddata | 14487073 | 12107325 | 11955690 | 11958819 | 12053579 | 11858107 |

**References**

1. Gunn D, Abbas Z, Harris HC, Major G, Hoad C, Gowland P, et al. Psyllium reduces inulin-induced colonic gas production in IBS: MRI and in vitro fermentation studies. Gut. 2021.

2. ZOE. Bake blue muffin 2021. Available from: <https://joinzoe.com/bluepoop/bake-blue-muffins>.

3. McCance RA, Widdowson EM. McCance and Widdowson's the Composition of Foods: Royal Society of Chemistry; 2014.

4. Svedlund J, Sjödin I, Dotevall G. GSRS—a clinical rating scale for gastrointestinal symptoms in patients with irritable bowel syndrome and peptic ulcer disease. Digestive diseases and sciences. 1988;33:129-34.

5. Williams BA, Bosch MW, Awati A, Konstantinov SR, Smidt H, Akkermans AD, et al. In vitro assessment of gastrointestinal tract (GIT) fermentation in pigs: Fermentable substrates and microbial activity. Animal Research. 2005;54(3):191-201.

6. Gunn D, Murthy R, Major G, Wilkinson-Smith V, Hoad C, Marciani L, et al. Contrasting effects of viscous and particulate fibers on colonic fermentation in vitro and in vivo, and their impact on intestinal water studied by MRI in a randomized trial. The American journal of clinical nutrition. 2020;112(3):595-602.

7. Rotbart A, Yao C, Ha N, Chrisp MD, Muir JG, Gibson PR, et al. Designing an in-vitro gas profiling system for human faecal samples. Sensors and Actuators B: Chemical. 2017;238:754-64.

8. Perez-Sepulveda BM, Heavens D, Pulford CV, Predeus AV, Low R, Webster H, et al. An accessible, efficient and global approach for the large-scale sequencing of bacterial genomes. Genome biology. 2021;22:1-18.

9. Blanco-Míguez A, Beghini F, Cumbo F, McIver LJ, Thompson KN, Zolfo M, et al. Extending and improving metagenomic taxonomic profiling with uncharacterized species using MetaPhlAn 4. Nature Biotechnology. 2023;41(11):1633-44.

10. McMurdie PJ, Holmes S. phyloseq: an R package for reproducible interactive analysis and graphics of microbiome census data. PloS one. 2013;8(4):e61217.

11. Smith S. phylosmith: an R-package for reproducible and efficient microbiome analysis with phyloseq-objects. Journal of Open Source Software. 2019;4(38).

12. Mallick H, Rahnavard A, McIver LJ, Ma S, Zhang Y, Nguyen LH, et al. Multivariable association discovery in population-scale meta-omics studies. PLoS computational biology. 2021;17(11):e1009442.

13. Barnett DJ, Arts IC, Penders J. microViz: an R package for microbiome data visualization and statistics. Journal of Open Source Software. 2021;6(63):3201.
